# *Ganoderma boninense* Disease Detection by Near-Infrared Spectroscopy Classification: A Review

**DOI:** 10.3390/s21093052

**Published:** 2021-04-27

**Authors:** Mas Ira Syafila Mohd Hilmi Tan, Mohd Faizal Jamlos, Ahmad Fairuz Omar, Fatimah Dzaharudin, Suramate Chalermwisutkul, Prayoot Akkaraekthalin

**Affiliations:** 1 College of Engineering, Universiti Malaysia Pahang, Gambang 26300, Malaysia; masira.hilmitan@gmail.com; 2 School of Physics, Universiti Sains Malaysia, Gelugor 11800, Malaysia; fairuz_omar@usm.my; 3 Department of Mechanical, Kuliyyah of Engineering, International Islamic University Malaysia, Jalan Gombak 53100, Malaysia; fatimahdz@iium.edu.my; 4 The Sirindhorn International Thai-German Graduate School of Engineering (TGGS), King Mongkut’s University of Technology North Bangkok, Bangkok 10800, Thailand; suramate.c.ce@tggs-bangkok.org; 5 Faculty of Engineering, King Mongkut’s University of Technology North Bangkok, Bangkok 10800, Thailand; prayoot.a@eng.kmutnb.ac.th

**Keywords:** oil palms, near-infrared spectroscopy, NIR spectrum ML classifier algorithms

## Abstract

*Ganoderma boninense* (*G. boninense*) infection reduces the productivity of oil palms and causes a serious threat to the palm oil industry. This catastrophic disease ultimately destroys the basal tissues of oil palm, causing the eventual death of the palm. Early detection of *G. boninense* is vital since there is no effective treatment to stop the continuing spread of the disease. This review describes past and future prospects of integrated research of near-infrared spectroscopy (NIRS), machine learning classification for predictive analytics and signal processing towards an early *G. boninense* detection system. This effort could reduce the cost of plantation management and avoid production losses. Remarkably, (i) spectroscopy techniques are more reliable than other detection techniques such as serological, molecular, biomarker-based sensor and imaging techniques in reactions with organic tissues, (ii) the NIR spectrum is more precise and sensitive to particular diseases, including *G. boninense*, compared to visible light and (iii) hand-held NIRS for in situ measurement is used to explore the efficacy of an early detection system in real time using ML classifier algorithms and a predictive analytics model. The non-destructive, environmentally friendly (no chemicals involved), mobile and sensitive leads the NIRS with ML and predictive analytics as a significant platform towards early detection of *G. boninense* in the future.

## 1. Introduction

The oil palm industry gives a major contribution to Malaysia’s economy and generates profitable export earnings for the country. In 2018, oil palm contributed 37.9% to the gross domestic product (GDP) of the agricultural sector [1]. The Malaysian Palm Oil Board (MPOB) reported that in 2019, Malaysia produced 17.18 tonnes per hectare of fresh oil palm fruit over 5.9 million hectares of the total planted area. Malaysia contributed 20.5% of world palm oil supplies, making Malaysia the world’s second-biggest palm oil manufacturer and exporter. Total export revenue was approximately RM 67.52 billion [2]. After 30 months of planting, oil palm trees begin to produce fruit and can bear fruits for 20 to 30 years. Oil palm is the world’s most efficient oil-bearing crop, which produces one tonne of oil in just 0.26 hectares of land (www.mpob.gov.my) (accessed on 30 January 2020).

Unfortunately, infection with *G. boninense*, a type of fungus, has caused great losses in the production of oil palm, which is of significant concern to the palm oil industry. Basal stem rot (BSR) disease caused by *G. boninense* can reduce the yield of oil palm production by 80% (estimated USD 28.4 billion/RM 117.6 billion). This disease is the main concern which badly affects Southeast Asia’s oil palm plantations, particularly in North Sumatra and Malaysia [3]. *G. boninense* can infect oil palm trees at all stages, from seedlings to mature plants [4]. This fungus is found to infect seedlings and trees less than a year old in the nursery [5,6] and spreads in the soil through roots and the air [7].

*G. boninense* is an unnoticeable necrotrophic fungus in the early stages of infection and forms uniform hyphae of infection within the host [8]. The fungus absorbs nutrients while producing enzymes and mycelia which degrade cell walls, thus generating the defense mechanism in the host plants [9]. The host cell dies in the final stage even before the Ganoderma fruiting bodies, basidiomata, are formed [10]. Secondary metabolites such as quinoline [11] are released in the tree within 24 h of a *G. boninense* infection to combat fungal incursion [12,13]. Quinoline belongs to the secondary metabolite alkaloid group and is derived from tryptophan, a precursor based on amino acids [14,15].

**Figure 1:**
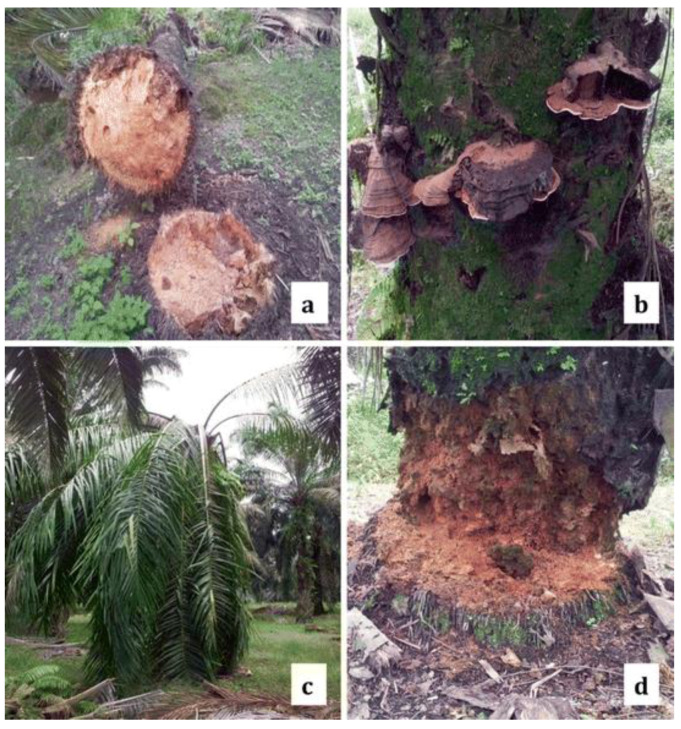
(**a**–**d**) Oil palm infected with *G. boninense* with (**a**) rotten bole tissue, (**b**) basidiomata of *G. boninense*, (**c**) foliar symptoms, (**d**) decaying oil palm bole tissues [20].

Various studies and approaches have been carried out to control BSR disease in oil palm trees but as yet there is no effective detection method for *G. boninense*. This failure has resulted in the death of oil palms due to the late detection of infection. When the disease symptoms begin to appear, more than half of the internal tissues are already rotten [16]. It takes 1 to 2 years for young palms to die from the onset of disease symptoms, while mature trees can live only up to 3 years [17]. The infection causes rotten internal tissues, which leads to stem fracture and the tree might collapse at any stage of the infection. The earliest visible external symptoms of BSR occur in the leafage, which are almost similar to the physical condition of water stress, malnutrition, hyperacid soil or high soil water salinity, as shown in Figure 1 [18,19]. It is therefore a physical diagnosis including a hyperspectral imaging method, but it is not effective.

Early detection and control strategies for *G. boninense* are still undeveloped, although it is identified as the major cause of death of oil palms. To date, removing the tree is an effective method for preventing BSR disease from spreading to others [21]. This is done through isolation processes of trenching, ploughing, harrowing, clearing, burning and fallowing before replanting the soil with seedlings [22]. Therefore, early detection and identification of *G. boninense* infection are very crucial to prevent production losses and reduce the cost of plantation management.

Table 1 presents several techniques of the early detection of plant disease. Visual inspection is used to evaluate the physical signs and symptoms of the plants. As reported in [23], this approach can detect a wide range of disease types. However, visual inspection of infected trees requires a great deal of labour and time [24].

Neither symptoms nor signs, however, provide accurate information. Therefore, to isolate and identify the causative agent, it may be necessary to bring a sample to the laboratory for further assessments. The first attempt to detect disease in plants is by using an enzyme-linked immunosorbent assay (ELISA) with polyclonal antibodies (PAbs) of the pathogen [35], and antibodies were employed to detect *G. boninense* in culture media [36]. Other lab-based techniques to detect BSR disease are Ganoderma selective medium (GSM) [37], multiplex PCR-DNA kits [38], GanoSken tomography [39] and electrochemical DNA biosensors [40]. This requires a massive workforce since the infected oil palm trunks are drilled for sampling and then *G. boninense* is nurtured in agar flats using semi-selective media [37]. On the other hand, direct molecular techniques, which involve preparing representative samples and extracting DNA, remain a challenge. These chemical-based techniques are tedious, complex, costly and time-consuming. Imaging and spectroscopy techniques are photonic techniques involving light–material interactions which allow the quantitative and qualitative analysis of agricultural products. Imaging techniques acquire spatial, colour and thermal information effectively, while spectroscopy techniques provide spectral information of the sample [41].

Recently, spectroscopy techniques to detect *G. boninense* have been explored. The majority of spectroscopic applications for the detection of plant diseases comply with the following criteria: non-invasive, rapid, sensitive and precise to particular diseases, which have been taken into consideration for the development and design of early stage infection detectors [42]. Spectroscopy techniques assess the condition of the plant by emitting visible and non-visible radiation at specific wavelengths to penetrate tissues and the backscattered light with certain intensities becomes an indicator of different conditions. These wavelengths are important for studying various plant fungal diseases [43].

There are several types of spectroscopy techniques, including visible (VIS), infrared (IR), nuclear magnetic resonance (NMR), mass spectroscopy (MS), impedance spectroscopy (IS), fluorescence spectroscopy (FS) and Raman spectroscopy (RS). NMR and MS belong to biomarker-based sensors which assess metabolite profiling of the plant. They can determine the chemical structures of molecules.

VIS/IR spectroscopy has higher accuracy than IS and FS in detecting plant disease. Additionally, VIS/IR spectroscopy is cheaper, easy to adapt, suitable for field measurements and able to provide early detection [32]. The VIS/IR wavelength is divided into four regions: visible (VIS), near-infrared (NIR), mid-infrared (MIR) and far-infrared (FIR) regions. NIRS has been used extensively for the rapid detection of organic components [44]. NIRS is often favoured over other spectroscopy and analytical methods as it has the highest accuracy for disease detection in different types of plants compared to mid-infrared (MIR) and visible to near-infrared (VIS-NIR) spectroscopy [45]. NIRS is more precise and sensitive to particular diseases, including *G. boninense*, compared to VIS light [46]. The VIS region provides information based on colour whereas the NIR region principally involves C-H, O-H and N-H vibrations. These vibrations contain information on the chemical elements, structures and states of molecules. For early asymptomatic disease detection, the NIR region is the main interest as NIR spectral data contain information on the interior tissue while VIS spectral data contain information on the exterior, such as colour and texture [47]. The shorter NIR wavelengths, compared to those in the MIR range, enable increased penetration depth and direct analysis of solid samples with minimal or no sample preparation [45]. The recent advancement in NIRS instruments is in on-site analysis with the availability of portable and compact instrumentation [48]. These advantages, along with being chemical free, rapid, non-destructive and non- invasive, make the utilisation of NIRS for a complete early detection system of *G. boninense* in real time possible. Raman spectroscopy (RS) involves the same complementary vibration spectroscopy technique as NIRS which also identifies vibrational transitions in molecules [49]. RS measures the scattering of light while NIRS measures the absorption of light [50]. RS needs a high concentration of the sample which makes it difficult to measure due to a low probability of Raman scattering. Photodegradation of the molecule may occur due to excitation of electronic absorption bands and the measurement may be disrupted due to the presence of fluorescence from impurities [41]. While RS is suitable for the measurement of moist samples, NIRS is suitable to measure the level of fluorophore contained in biosamples. This makes NIRS more applicable to the measurement of plants and plant-related matter [51]. 

This review shows the potential of NIRS for disease prediction, coupled with classification techniques, as a convincing rapid analytical tool for the early detection of *G. boninense* in oil palm. A previous study based on spectroscopy techniques for *G. boninense* detection will be discussed in Section 2. Section 3 will deliberate on the theory, principle, advantages and disadvantages of NIRS. The application of NIRS on the detection of plant diseases is discussed in Section 4. Meanwhile, Section 5 discusses several machine learning techniques for plant disease prediction, which include k-nearest neighbour (kNN), naïve Bayes (NB), decision tree (DT), artificial neural network (ANN) and support vector machine (SVM).

## 2. Spectroscopy Technique for *Ganoderma boninense* Detection

Several spectroscopy techniques have been conducted to detect the infection of *G. boninense* in oil palms, such as MS and NMR spectroscopy [52,53], dielectric spectroscopy [54,55], Fourier transform Infrared (FTIR) spectroscopy [56,57,58], MIR spectroscopy [59], hyperspectral imaging spectroscopy [60] and visible to near-infrared (VIS-NIR) spectroscopy [46,61].

Metabolite profiling of *G. boninense* is assessed by using MS and NMR spectroscopy [52,53]. Isha et al. [52] used the MS approach on oil palm root while they [53] also used the MS approach on oil palm leaves to identify the metabolite variation of *G. boninense*-infected and non-infected plants. Both studies employed PCA to discriminate between the infected and non-infected samples. A study by Khaled et al. [54] employed dielectric spectroscopy using impedance, capacitance, dielectric constant and dissipation factors for early detection of *G. boninense* in oil palm. Dielectric spectroscopy (DS), also known as impedance spectroscopy (IS), operates in the radio and microwave frequency ranges of the electromagnetic spectrum [62]. The impedance values produced the most significant classification between healthy samples and different levels of *G. boninense*-infected samples. Accuracies up to 80% are achieved by implementing SVM and ANN. SVM produces better classification accuracy than ANN. A similar study using dielectric spectroscopy to detect *G. boninense* by Khaled et al. [55] implemented discriminant analysis (LDA), quadratic discriminant analysis (QDA), k-nearest neighbour (kNN) and naïve Bayes (NB) classifiers to classify the oil palm samples based on the level of infection. The impedance values produced the most significant classification with 95.45% accuracy. The mean accuracies of the dielectric properties were 80.34%, 80.79%, 77.85% and 79.98% by using LDA, QDA, kNN and NB, respectively.

**Figure 2:**
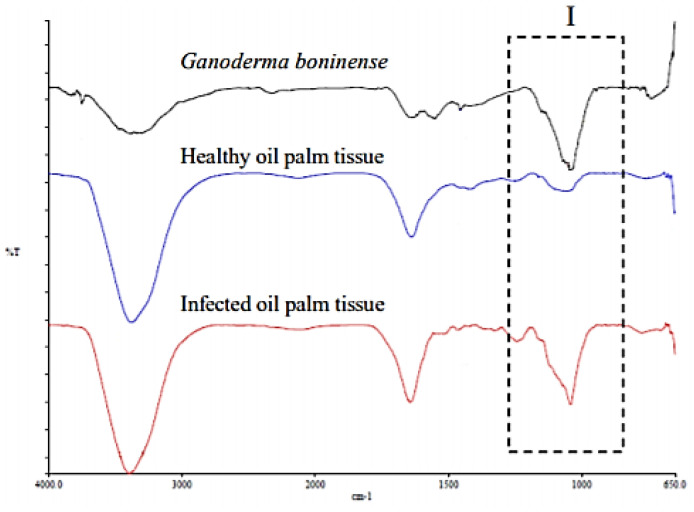
FTIR spectra of *G. boninense*, healthy oil palm tissue and infected oil palm tissue [56].

Dayou et al. [56] investigated the possibility of using the FTIR spectroscopy technique to detect *G. boninense* infection and to distinguish between healthy and infected oil palm trunk tissue. The results were evaluated based on the FTIR spectra pattern. The significant resemblance of the infected oil palm tissue and pure *G. boninense* compared to the healthy sample can be observed in region I of the FTIR spectra illustrated in Figure 2. They can be used as biomarkers for *G. boninense* detection. This finding corroborates the study in [63], which reported a unique IR pattern due to the presence of fungi to discriminate between infected tissues and uninfected tissues. A similar study by Alexander et al. [57] reported that FTIR spectroscopy is capable of detecting *G. boninense* infection contents as low as 5%. In addition, FTIR spectroscopy is able to identify the functional group of *G. boninense*. A study by Abdullah et al. [58] identified CH_3_, CN and C-O-C in the *G. boninense* fruiting body. On the other hand, Arnnyitte et al. [64] identified the N-H, C=N, C=H and C-O-C functional groups present in *G. boninense*-infected oil palm tissue, which are absent in healthy oil palm tissue. These significant results represent reliable discrimination between infected and healthy oil palm samples.

The feasibility of MIR spectroscopy for *G. boninense* detection by using an FTIR spectrometer is assessed by Liaghat et al. [59]. Oil palm leaf samples were ground into powder and processed into pellets for the spectroscopy measurement. In this study, linear discriminant analysis (LDA), quadratic discriminant analysis (QDA), k-nearest neighbour (kNN) and naïve Bayes (NB) classifiers were used to classify different levels of disease severity. The LDA classifier showed the highest overall classification accuracy of 92%. This study shows that MIR spectroscopy, along with the classification approaches, is able to detect and differentiate the level of *G. boninense* infection [59].

Shafri et al. [60] applied VIS-NIR spectroscopy (350–1000 nm) for *G. boninense* detection using a hyperspectral remote sensing instrument and a portable spectroradiometer. The spectral differences between healthy and infected leaves of 6-month-old oil palm seedlings were identified. Three levels of disease severity, healthy, mild and severe, could be identified from the spectral reflectance. Classification of the severity level was performed using a maximum likelihood classifier based on the most significant spectral wavelength. The net accuracy was found to be 82%. Ahmadi et al. [61] utilised VIS-NIR spectroscopy (273–1100 nm) with a portable spectroradiometer to discriminate and classify *G. boninense* infection levels in oil palm trees at an early stage. The samples were classified based on the level of severity: healthy, mild, moderate and severe. Accuracy up to 100% was achieved by applying an artificial neural network (ANN) classifier on the raw spectral data without any pre-processing approaches. Lelong et al. [65] utilised VIS-NIR spectroscopy (310–1130 nm) to evaluate healthy trees and several *G. boninense*-infected oil palm levels based on the hyperspectral reflectance data. A classification accuracy of 94% was achieved by using PLS-DA.

**Figure 3:**
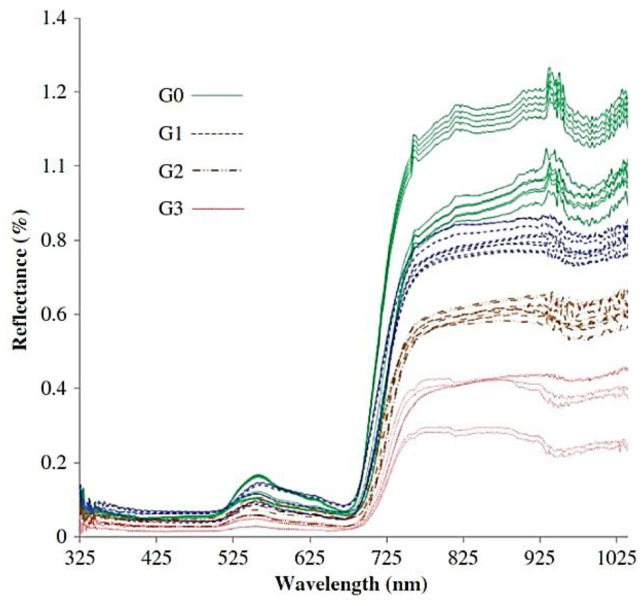
Reflectance spectra of healthy (G0) and non-healthy (G1, G2 and G3) leaf samples [46].

Another similar study by Liaghat et al. [46] assessed in-field VIS-NIR spectroscopy (325–1075 nm) to detect *G. boninense* infection in oil palm. Significant differences between each severity level in the NIR region compared to the VIS region are shown in Figure 3, which depicts the ability of NIRS to detect *G. boninense*. The reflection in the NIR region decreases drastically with increasing disease severity while the healthy leaves show the highest reflectance. Such results are possible due to the degradation of cell walls or wilting in plants [66]. Similar classification techniques [59] have been utilised to discriminate four levels of disease severity. kNN was revealed to be the best classifier with the highest classification accuracy of 97.3% compared to other models. The classifier could differentiate the level of severities of Ganoderma-infected oil palm from healthy ones [46].

These numerous studies demonstrate the ability of spectroscopy techniques paired with classification algorithms, which has led to promising results for the detection of *G. boninense* infection, as summarised in Table 2. Thus, future research on the detection of *G. boninense* using these approaches should be conducted more extensively. Despite the fact that NMR, MS, DS and FTIR spectroscopy approaches are able to discriminate infected and the healthy oil palm, these techniques were carried out under laboratory conditions, so they are impractical for real-time in-field measurements. These techniques are destructive since the samples need to be processed prior to measurements. FTIR spectrometers used to perform FTIR and MIR spectroscopic analysis are bulky in size, forcing the measurement to be performed in the laboratory. Additionally, FTIR requires the samples to be processed into pellets before the lab measurements.

VIS/IR spectroscopy has higher accuracy in detecting plant disease than the other spectroscopy methods [32]. Based on the VIS-NIR spectroscopy study in [46], the spectral data in the NIR region portray significant differences between classes of samples compared to the VIS region. Liang et al. [67] stated that the VIS region is only useful for visual analysis; thus, it is not useful for asymptomatic detection. Therefore, further research to detect *G. boninense* based on NIRS alone without coupling with VIS spectroscopy should be considered for the early stage of infection where there is no visible symptom of infection. From the findings of Abdullah et al. [58] and Arnnyitte et al. [64], the functional groups of *G. boninense* are CH_3_, CN, N-H, C=N and C-O-C. Several functional groups can also be identified in the NIR region, such as CH_3_, N-H and C=H [68]. NIRS demonstrates capability of detecting *G. boninense*, therefore, a higher accuracy NIR sensor is demanded to gain a better *G. boninense* detection rate. NIR instruments are available in portable compact versions, thus, a rapid on-site analysis can be performed directly. It is noticed that the recent portable DLP NIRscan Nano evaluation module (EVM) produced by Texas Instruments has been used in detecting organic compounds. This affordable miniature sensor allows higher performance measurements to be made, thus it can be a great potential sensor to detect *G. boninense*. The theory and operating principle of NIRS, followed by the advantages and disadvantages of NIRS, are discussed in the next section. 

## 3. Near-Infrared Spectroscopy

### 3.1. Theory and Operating Principle

**Figure 4:**
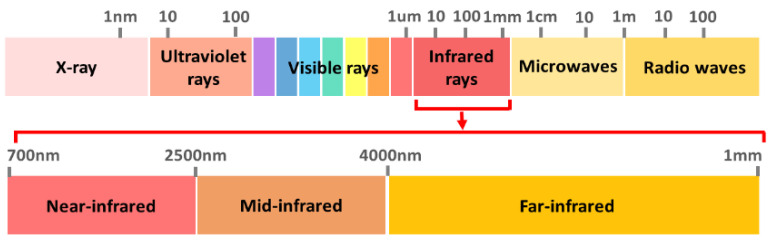
The electromagnetic spectrum.

NIRS is a spectroscopic method that operates in the NIR region from 700 to 2500 nm (430–120 THz), as shown in Figure 4. The sample is illuminated with a broad spectrum of the NIR operating wavelength, which can be absorbed, transmitted, reflected or scattered by the targeted sample. A spectrum is produced by absorbed light based on vibration frequencies of molecules in the sample [69]. The collected spectrum gives information on the properties of organic molecules in the sample which is related to the molecular composition.

The energy absorbed by a sample in the NIR region causes the covalent bond to vibrate between oxygen and hydrogen (O-H), carbon and hydrogen (C-H) and nitrogen and hydrogen (N-H), resulting in NIR absorbance bands [70]. Consequently, most chemical and biochemical species have unique absorption bands that can be used for qualitative and quantitative analysis. The shorter wavelengths weaken the intensity of bands. The weak band intensities in the NIR region mean that the solid samples do not need dilution and has a minimum non-linearity effect [71]. Three important and diagnostic functional groups of NIR absorption can be found in organic compounds, as indicated in Table 3.

NIRS measurements can be collected in two modes, either transmittance/absorption or diffuse reflectance [72]. Transmittance is measured on translucent samples while diffuse reflectance is measured on opaque or light-scattering matrices [68]. In transmission mode, incident light irradiates on the side of the sample, traverses into the pore structure and the transmitted light is detected on the other side of the sample. Whereas in diffuse reflection, light illuminates the surface of the sample, is diffusely reflected from the sample surface and then detected [73].

**Figure 5:**
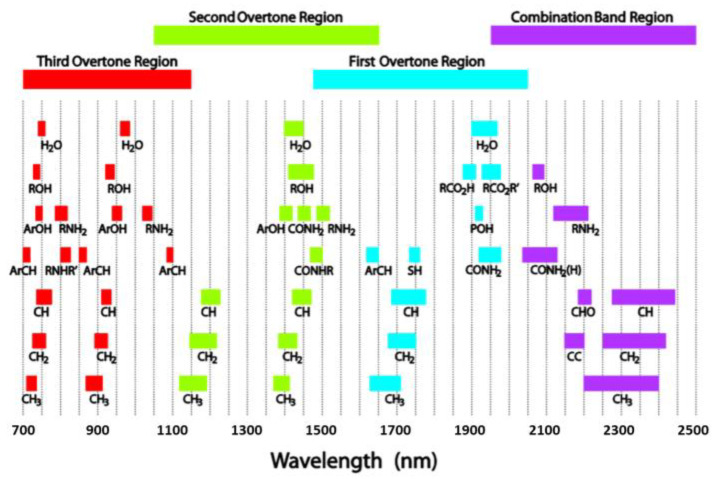
Major analytical bands and relative peak positions for prominent NIR absorptions [68].

Light is absorbed corresponding to the combinations and overtones of vibrational frequencies of the molecules in the sample. Overtones can be considered as harmonics because at multiple frequencies they produce a series of absorptions. Overtones appear when a vibrational mode is excited at a higher frequency than the fundamental vibration. Overtone stretching involves a change of the bond length and bending, involving a shift in the angle of two bonds. Combinations, on the other hand, are much more complex. NIR absorption is in a higher state of excitation, so it requires more energy than fundamental absorption. The combinations between two or more basic absorptions appear from the sharing of NIR energy. There will be a very large number of combinations when the number of overtones in a molecule from a group of fundamental absorptions is small [74]. Figure 5 shows the major overtones and combinations observed in the NIR spectral region.

Although samples with different organic compositions produce unique spectra, the overlapping spectral bands containing peaks, valleys and curvature complicate the spectra interpretation [75]. Specific data analysis to relate the spectral data with the physical and chemical composition is required to interpret these absorption bands. In order to extract the relevant information, calibration of NIR spectra must be performed [76]. A mathematical relationship between the two datasets, including physical or chemical product information, should be established to perform calibration.

### 3.2. Advantages of Near-Infrared Spectroscopy

NIRS has tremendous potential for various agricultural applications such as determination of soil content [77] and fruit quality [78] and detection of plant disease [79] and fungal infection [80]. It is a non-destructive analytical technology which provides rapid and accurate analysis. NIRS is a reliable and non-invasive technique with potential for plant disease detection. Detection and quantification of endophyte alkaloids in perennial ryegrass has been performed by utilising NIRS [81]. From this study, NIRS was able to assess secondary metabolites (i.e., alkaloids) in viable plant tissues. Since infected oil palm trees release secondary metabolites (i.e., quinoline) that belong to the alkaloids [11,14], there is the possibility to detect infection before symptoms appear.

It is an environmentally friendly technique as there is no chemical needed for this method; no disposal of chemicals is involved [82]. Thus, the detection of *G. boninense* in palm oil can be conducted without affecting the samples. NIRS is also capable of handling the bulk of data measurement of inhomogeneous samples. Moreover, minimal or zero sample preparation is required before NIRS measurement, which saves time and cost [83]. The rapid measurement of NIRS along with these other advantages make NIRS fit for automatic and online analysis for routine procedures [84].

Furthermore, NIRS has the highest accuracy for disease detection in different types of plants compared to MIR and VIS-NIR spectroscopy, as summarised in Table 4. NIR has a shorter wavelength, and thus has a higher penetration depth into samples compared to MIR [45]. NIRS is more precise and sensitive to particular diseases, including *G. boninense*, compared to VIS light [45]. NIRS also suitable for the early detection of plant disease as it is associated with the interior information of the sample [47].

### 3.3. Disadvantages of Near-Infrared Spectroscopy

NIRS requires data from the golden method (i.e., chemical analysis) for calibration purposes which requires a number of samples with known analyte concentration to validate the samples [85]. Calibration involves abundant sample data and complex analysis, which is necessary to determine the relationship between the spectral and golden method. The predictive accuracy of NIRS depends on the reliability and accuracy of the calibration. Once calibrated, the measurement of future samples can be easily measured and analysed by the calibration model to identify the spectral composition of the samples. This will reduce time and cost in the long term. However, measurements beyond the sample calibration range are invalid [85]. The NIR spectra often overlap, thus data quantification and interpretation are challenging and require significant time, resources and money [86].

## 4. Application of NIRS for Plant Disease Detection

Having discussed NIRS in the previous section, this section will review the literature on NIRS for plant disease detection. These diseases include: fungal contamination in mushrooms [87], begomovirus disease on papaya leaves [79], zebra chip disease in potato [88], stripe rust on wheat plants [89], bitter pit in Honeycrisp apple [90], anthracnose disease and fruit fly eggs and larval infestation in mango [91,92], fungal infection in maize [93,94,95], chestnut [96], barley [80] and almond kernel [67], leaf miner infestation in tomato leaves [97], Fiji leaf gall disease in sugarcane [98] and mycotoxin contamination in rice [99] and red paprika [100], as summarised in Table 5.

The most recent study by Wang et al. [87] applied NIRS, MIRS and an electronic nose (E-nose) to detect the fungal contamination of freeze-dried edible mushrooms, Agaricus bisporus. Partial least squares discriminant analysis (PLS-DA) was used to classify the samples. Remarkably, NIRS outperformed the other two methods by achieving the highest overall accuracy of 99% for discrimination of fungal species and 99.2% for each storage period. A study by Haq et al. [79] detected begomovirus infection on papaya leaves using two reflectance spectroscopy approaches, NIRS and FTIR with attenuated total reflection. Both spectroscopy techniques with the aid of PLS-DA were capable of in vivo detection of begomovirus infection. NIRS has also been utilised to detect zebra chip disease in potatoes [88]. Canonical DA was utilised to classify the infected potatoes from non-infected potatoes with 98.35% and 97.25% total classification accuracy on raw spectra and 2nd derivative spectra, respectively.

Another study by Zhao et al. [89] applied NIRS to quantitatively detect stripe rust disease on wheat caused by Puccinia striformis f. sp. tritici (Pst) in the incubation period. This study claims that the detection of DNA of Pst in leaves during the incubation period could also be fulfilled using NIRS. Three classification models were utilised: quantitative partial least squares (QPLS), support vector regression (SVR) and the integration of both QPLS and SVR. All models produced R^2^ values of the training set and the testing set of more than 0.5 which demonstrated that there is a relatively high correlation between the NIR spectral absorbance and the content of Pst DNA in wheat leaves. NIRS has been evaluated for bitter pit detection in Honeycrisp apple [90]. A spectroradiometer in the range of 300 to 2500 nm, which is in the VIS to NIR region, was utilised. However, only the NIR region (800 to 2500 nm) was taken into consideration for the analysis and classification. QDA and SVM were applied on the spectral data with overall classification accuracy in the range of 73–96% and 69–89%, respectively.

NIRS was applied on mango fruits to detect anthracnose disease [91]. The classification accuracy of artificially infected mangoes and non-infected mangoes was 89% using PLS-DA. In addition, NIRS was also utilised to detect fruit fly eggs and larval infestation in intact mango fruits [92]. Two modes of NIRS were used: interactance mode (700–1100 nm) and reflectance mode (1100–2500 nm). PLS-DA was implemented to classify infested mango and non-infested mango. The standard deviations (SDs) of the predicted class value in interactance mode were 0.27 for infested mango and 0.19 for non-infested mango. Meanwhile, in reflectance mode, the SD value was 0.26 for infested mango and 0.28 for non-infested mango.

A NIRS technique was utilised to detect fungal infection in maize kernels [93,94,95]. kNN classification used in [93] at two NIR wavelengths (i.e., 715 and 965 nm) provided correct classification of healthy and infected kernels with an accuracy of 98.1% and 96.6%, respectively. A similar study by Draganova et al. [94] classified healthy and Fusarium fungus-infected maize grains by using soft independent modelling by class analogy (SIMCA), a probabilistic neural network (PNN) and k-means classifier. The PNN produced the best performance, with an accuracy of 99.3% and 98.7% for healthy and diseased grains, respectively. A study by Tallada et al. [95] discriminated eight fungus species at different levels of infection: asymptomatic, mild, moderate and severe. Linear and non-linear prediction models from the NIR spectra were developed using LDA and multi-layer perceptron (MLP) neural networks. The results for detecting all levels of infection were 89% for uninfected kernels and 79% for infected kernels.

In [96], the authors applied NIRS for the detection of fungal infection in chestnuts. LDA, QDA and kNN classifiers were applied to classify healthy chestnut and medium and severely infected chestnut. The study reveals that NIRS shows the feasibility of detecting the separation between healthy and infected chestnut, with the highest classification accuracy of 97% using QDA. The application of NIRS to identify Fusarium fungi in barley was investigated in [80]. PLS-DA was used for discriminant prediction of normal hulled barley and Fusarium-infected hulled barley. A classification accuracy up to 100% was achieved.

The potential of NIRS to detect fungal infection in almond kernels caused by Aspergillus flavus (A. flavus) and Aspergillus parasiticus (A. parasiticus) was investigated by [67]. Canodical discriminant analysis (CDA) was applied to the NIR spectra with a total cross-validation error rate of 0.26% and zero false-negative errors. The authors decided to exclude the VIS spectra (below 800 nm) for model development since the discrimination of the infected and uninfected almond kernels did not involve analysing visual differences of the kernels.

A study by Xu et al. [97] was conducted to assess NIRS for the detection of leaf miner infestation in tomato leaves. Reflectance spectra of tomato leaves at various levels of infection were characterised. Significant differences in reflectance among infestations were observed at the wavelengths of 1450 nm and 1900 nm, which was useful to discriminate levels of leaf miner infestation. Regression analysis for predictive modelling was performed on both wavelengths. A single wavelength reflectance at 1450 nm showed a good prediction performance of R^2^ = 0.982. In addition, NIRS was implemented for the determination and rating of sugarcane resistance against Australian sugarcane disease and Fiji leaf gall [98]. Partial least squares (PLS) regression was performed on the NIR spectra. Adequate results for the standard error of validation (SEV) and SEP of 0.98 (R^2^ = 0.97) and 1.20 (R^2^ = 0.88) were recorded, respectively.

NIRS is proven to be feasible in detecting mycotoxins such as aflatoxin and ochratoxins [99,100]. Mycotoxins are toxic secondary metabolites produced by fungi. Aflatoxin B1 (AFB_1_) contamination in rice samples was identified by using NIRS [99]. Partial least squares (PLS) regression calibration models constructed from healthy and infected plants were based on NIR spectra. A correlation of 0.850 and a standard error of prediction (SEP) of 3.211% were achieved which revealed that NIRS has the ability to detect aflatoxin B1 in rice. Utilisation of NIRS for the determination of AFB_1_, ochratoxin A (OTA) and total aflatoxins in red paprika was also investigated [100]. Modified PLS (MPLS) was applied for the estimation of AFB_1_ (R^2^ = 0.95), OTA (R^2^ = 0.85) and total aflatoxins (R^2^ = 0.93).

These numerous studies thus far provide evidence that NIRS has tremendous capability to detect various plant diseases, including secondary metabolite incursions. Additionally, the ability of NIRS to detect secondary metabolites such as aflatoxins and achratoxins [99,100] demonstrates that NIRS might also be able to detect quinoline, a secondary metabolite produced by *G. boninense* [11]. To sum up this section, NIRS with the aid of machine learning and statistical approaches is a reliable tool for disease monitoring and early detection of plant diseases.

## 5. Machine Learning Techniques for Plant Disease Prediction

In most literature, various approaches and techniques have been utilised to analyse spectral data for plant disease detection, as stated in Table 2 and Table 3. Implementation of machine learning algorithms for disease detection are in contrast with the traditional system as it delivers decisive information and enables prediction of the upcoming outcome. Predictions cannot be made directly from the spectral data. Thus, machine learning is required to establish a prediction model. Machine learning discerns data patterns by extracting information from a dataset and transforming it into useful data to assist user decision making. It has gained interest in current agricultural technologies as a promising approach for faster and efficient data analytics [101]. Several algorithms were chosen and evaluated to in deploying a reliable and accurate prediction. Predictive modelling used to predict plant disease is related to several machine learning tasks, such as classification, regression and clustering [102]. To predict whether a plant is healthy or infected, disease prediction of plants based on a classification technique should be applied on the spectral data.

**Figure 6:**
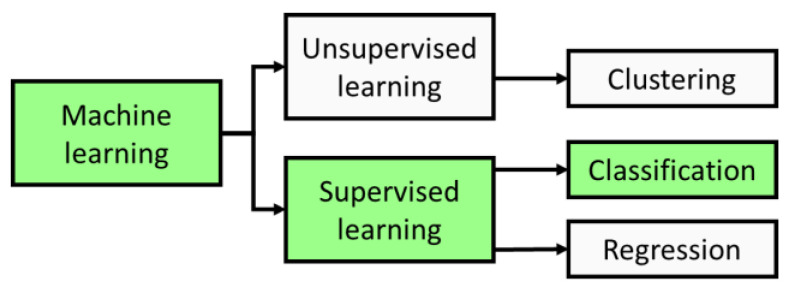
Main types of machine learning.

There are two main types of machine learning: unsupervised and supervised learning, as illustrated in Figure 6. Unsupervised learning methods denote a dataset without ground truth labels. These methods are capable of calculating linear and non-linear models with few statistical assumptions, and flexibly adapting to an extensive range of data features [103]. In contrast to unsupervised learning, supervised learning is primarily based on the data provided by a set of samples which is supposed to correctly represent all the related classes [104]. A supervised learning algorithm uses a known set of input data and known output responses and trains a model to generate accurate predictions of new data. This review only describes a supervised classifier as supervised classification algorithms create a model based on a training dataset for predicting unlabelled or new data, which is convenient for plant disease detection and prediction systems.

**Figure 7:**
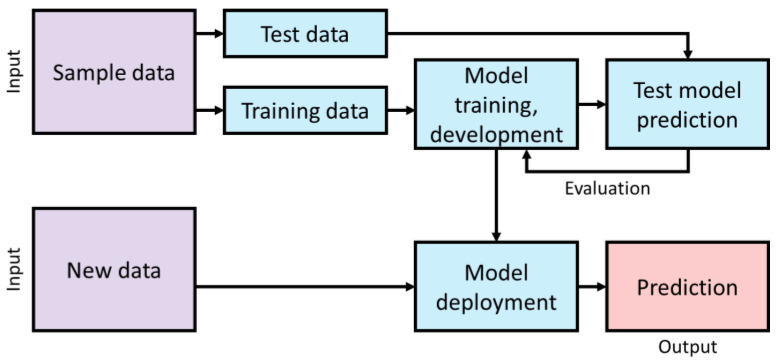
Machine learning model development.

Generally, machine learning classifiers are used to classify each item in a set of data into one of a predefined set of classes [105]. Classification is applied for pattern and object recognition based on features [106]. It is the process of determining the class of the input database in a training set of data to predict the qualitative target. The development of an ML classification model for prediction is illustrated in Figure 7.

The sample data are split into two: test data and training data. The training set is randomly sampled from the dataset whereas the remaining data form the test set. In the learning step, the classification model is developed by analysing the training data, whereas, in the classification step, the class labels for given data are predicted. Testing data are used to assess the performance of the classifier as a predictor to verify its applicability [107]. In order to acquire a good classification model, different classification algorithms should be tested out for assessment of the performance. Then, we evaluate the established model and deploy it for prediction. Several significant supervised classification algorithms along with their applications in plant disease detection are described in this section of the review paper:

- k-nearest neighbour (kNN);
- Naïve Bayes (NB);
- Decision tree (DT)—random forest and decision forest;
- Artificial neural network (ANN);
- Support vector machine (SVM).

kNN is a simple classifier which is widely used for pattern recognition. It is a lazy learning method based on learning which compares a given test sample with the available training samples [108]. Its simplicity enables ease of classification [109]. Classification is achieved by (i) identifying the nearest neighbours of the trained data, (ii) calculating the distance between them and input data and (iii) predicting the class of input data [110]. This classifier is suitable to be implemented on multi-modal classes in which a sample can have many class labels [111]. Liaghat et al. [46,59] employed a kNN classifier for *G. boninense* detection that classified four different classes of palm oil health conditions and generated the highest classification accuracy of 97.3%. kNN has also been implemented on NIR spectra to classify the severity of fungal infection in maize [93] and chestnuts [96].

NB is a simple Bayesian probabilistic classifier based on the Bayes decision theorem. The Bayes theorem is strong independence assumption theorem [112]. This assumption is considered naïve as it assumes that the effect of a feature on a class is not statistically influenced by the other features [113,114]. NB enables the prediction of class membership probabilities which determine the probability that a given data item belongs to a particular class label [115]. NB has been increasingly applied for classification due to its efficiency, simplicity and good performance. Implementation of NB on spectral data has been tested for the detection of *G. boninense* [46,59]. However, NB proved to have the lowest average overall classification accuracy compared to LDA, QDA and kNN. A study by Thakur and Mehta [116] successfully applied NB for the classification of disease in apple and mango. They also claimed that NB performed better than ANN in terms of precision and implementation speed.

A decision tree (DT) classifier is a predictive model which maps observations of data for the determination of the class of a given feature [115]. It has a tree-like structure in which all sources are split into subsets based on their attribute values [117]. Class labels are represented by the leaves and conjunctions of features leading to those classes are represented by the branches. This process splits the data until no further splitting is possible or all have the same value of the target variable. Most decision trees consist of a random forest tree classifier which outputs the category based on classes by a particular tree [118]. Sankaran et al. [119] investigated VIS-NIR spectroscopy as an approach to detect laurel wilt disease on avocado leaves by introducing four different classifiers, including a DT-based classifier. DT yielded high classification accuracies of over 94% when classifying asymptomatic leaves from infected plants.

ANNs or neural networks (NNs) imitate human brain function which enables them to complete complex tasks such as pattern generation, cognition, learning and decision making [120]. ANN is a conventional compact model representation for the analysis of high-dimensional data [121]. The input and output are represented by nodes, inspired by the concept of the biological neuron system [122]. There are three layers of nodes: input layer, hidden layer and output layer. The interconnected processing units are organised in a specific topology. Data enter the system via the input layer and learning occurs in one or more hidden layers while the decision or prediction is fulfilled through the output layer [103]. As mentioned previously in Section 2, Ahmadi et al. [61] successfully implemented ANN on VIS-NIR spectra for early prediction of *G. boninense* in oil palm with an accuracy of up to 100%. Another early detection of Botrytis cinerea (B. cinerea) on eggplant leaves by ANN is demonstrated in [47]. The developed ANN model based on the VIS-NIR spectral data has successfully predicted B. cinerea infection with an accuracy of 85% even before the presence of visible symptoms on leaves [47].

The SVM has been used in many applications as this classifier is effective and robust to noise [123]. The SVM was initially intended for binary classification and was investigated in order to solve multi-class classification problems. It allows the SVM to classify sample into two classes or more. SVM constructs or locates the optimal hyperplane as the decision line, separating the positive (+1) classes from the negative (−1) classes in the binary classification with the two classes’ largest margin [124]. If the samples are linearly separable, the SVM is used to find the optimal separating hyperplane. This is done by maximising the margin between the hyperplane and the training sample, called support vectors [123,125]. SVM was successfully applied to detect and classify grape leaf diseases with an accuracy of 88.89% [126]. In another study, SVM was applied to hyperspectral reflectance data to discriminate healthy and infected sugar beet leaves. A classification accuracy up to 97% was obtained [127].

This section has demonstrated the applications of several machine learning algorithms for early detection of plant diseases based on spectral characteristics. Machine learning has also been employed on oil palm spectroscopy data for classification of *G. boninense* in oil palm [46,54,55,59,60,61,65]. Each classifier has its benefits and drawbacks, as summarised in Table 6 [112,128]. However, the performance of classifiers is mostly influenced by the nature of the dataset. Thus, the comparison of classifiers of the measured data must be assessed before developing a complete predictive model.

## 6. Challenges and Future Prospects

This review paper revealed the prospects of NIRS in conjunction with machine learning algorithms for detecting different diseases and health conditions in plants. This review also revealed that a spectral-based classification approach has important implications for *G. boninense* infection. Thus, it can be concluded that NIRS with the aid of a machine learning classifier has a vast potential for early detection of *G. boninense* in oil palm. However, there are several challenges in the development of NIRS detection approaches. NIRS is a non-trivial process since it requires extensive interpretation of spectral data.

Moreover, variables such as sample size, temperature and humidity should also be taken into account during sample preparation or collection to standardise measurement. Environmental conditions can influence the spectral result of the sample [129]. In addition, NIRS spectral data depend on the large scale of reference methods for calibration. Thus, the accomplishment of accurate laboratory or chemical tests is crucial to verify the condition of the sample. As for the development of a predictive model, fundamental knowledge about related machine learning classifiers is desired. A comparative study needs to be done to select the best classifier for the final predictive model.

**Figure 8:**
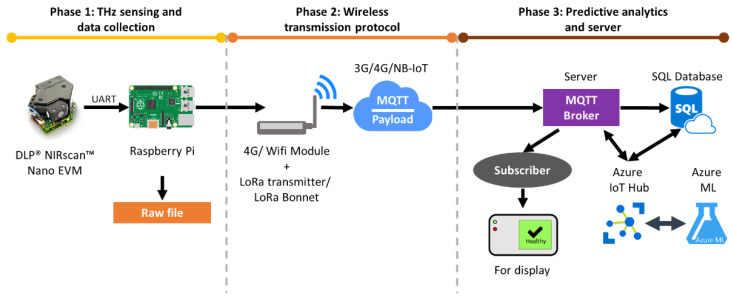
Proposed method of real-time NIRS *G. boninense* detection.

The reviews on the capability of NIRS to detect *G. boninense* have been comprehensively discussed, yet there is no further development of NIRS techniques for early detection of *G. boninense* infection in real time. Most of the deployments of NIRS for *G. boninense* detection are still conducted manually or offline. Therefore, real-time detection of *G. boninense* by using a portable NIR spectrometer, such as a DLP NIRscan Nano EVM, is anticipated, as shown in Figure 8. This proposed work is a complete system of NIRS real-time feedback with online classification, a combination of which allows rapid and accurate recognition between healthy and infected palm oil trees. The NIR spectrometer is small and portable which is very convenient for on-site real-time measurement. The proposed research involves an Internet of Things (IoT)-based NIRS predictive analytics system. First, the spectral data are acquired using the NIR spectrometer via a Raspberry Pi 3. An MQTT server and cloud connector are embedded in the Raspberry Pi 3 to connect the proposed prototype to the cloud for web-based configuration management, which requires a LoRa transmitter and 4G/wireless module to connect physically to the Raspberry Pi 3 for wireless data transmission. The server is established to enable access via several devices such as mobile phones, laptops or tablets for ease of monitoring of the analysis. The real-time detection system is built using the cloud platform Microsoft Azure and a Raspberry Pi 3. The spectral data are transferred to the Azure IoT Hub to undergo ML classification. Then, the data are streamed to an SQL database. The result is then visualised and stored in a database for future analysis. All raw values are stored in a database and can be used for the prediction of new oil palm samples. This proposed system enables real-time detection of *G. boninense* at the early stage of infection, which is a clear improvement on current methods.

## 7. Conclusions

This review paper demonstrates the utilisation of NIRS techniques along with a machine learning classifier as a feasible method for early detection of *G. boninense* in oil palm. Most of the studies only focus on detection without further development of the whole system. In Section 2, a review on spectroscopy techniques for *G. boninense* detection is presented. Spectroscopy techniques have been successfully implemented for *G. boninense* detection, but the studies were mostly conducted in the laboratory and based on manual analysis of the spectra. It is found that NIRS is applicable instead of VIS spectroscopy and other spectroscopy techniques for *G. boninense* detection. A suitable machine learning classifier model based on kNN, ANN, NB or SVM must be executed with the NIR spectra as input data to predict and classify healthy and infected oil palm, as explained in Section 5. Nonetheless, it is identified that kNN is a potential classifier for this research prototype since kNN is easy to be implemented and exhibits decent performance for prediction. With the advancement of this field, portable NIRS devices could be used commercially in the near future for a diagnosis technique and for other applications.

## Figures and Tables

**Table 1:** Methods for plant disease detection.

Disease Detection in Plants				
Physical Inspection	Serological Methods	Molecular Methods	Biomarker-Based Sensors	Remote Sensing
Visually, based on external symptoms [24]	Flow cytometry [25]	Fluorescence in situ hybridisation (FISH) [26]	Gaseous metabolite profiling [27]	Imaging techniques [28]
	Enzyme-linked immunosorbent assay (ELISA) [29]	Polymerase chain reaction (PCR) [30]	Plant metabolite profiling [31]	Spectroscopy techniques [32]
	Immunofluorescence [33]	DNA arrays [34]		

**Table 2:** Previous studies on *G. boninense* detection using spectroscopy techniques.

Spectroscopy Method	Instrument	Sample Grouping	Models/Algorithms	Significant Result	References
Dielectric spectroscopy	Solid dielectric test fixture + impedance analyser	Healthy Mild Moderate Severe	SVM, ANN	Overall classification accuracies of the impedance values are more than 80%	[54]
		Healthy Mild Moderate Severe	LDA, QDA, kNN and NB	Mean classification accuracy: LDA: 80.34% QDA: 80.79% kNN: 77.85% NB: 79.98% Impedance value overall accuracy: 95.45%	[55]
Mass spectroscopy	GC-MS	Healthy Infected	PCA	The metabolite variation of healthy and infected oil palm root is identified	[52]
NMR spectroscopy	NMR spectrometer	Healthy Infected	PCA	The metabolite variation of healthy and infected oil palm leaves is identified	[53]
FTIR spectroscopy	FTIR spectrometer	Ganoderma basidiomata	-	CH_3_, CN and C-O-C functional groups are identified in the *G. boninense* basidiomata tissue.	[58]
FTIR spectroscopy	FTIR spectrometer	Healthy Infected	-	Resemblance pattern of infected oil palm with pure *G. boninense* is observed at a particular wavelength which can be used as biomarker	[56]
				*G. boninense* contents as low as 5% were detected	[57]
				N-H, C=N, C=H and C-O-C functional groups are identified in the *G. boninense* infected oil palm tissue	[64]
MIR spectroscopy	FTIR Spectrometer	Healthy Mild Moderate Severe	LDA, QDA, kNN and NB	The highest overall classification performance using LDA: 92% accuracy	[59]
VIS-NIR spectroscopy	Spectroradiometer	Healthy Mild Severe	Maximum likelihood	82% classification accuracy	[60]
VIS-NIR spectroscopy	Spectroradiometer	Healthy Mild Moderate Severe	ANN	Up to 100% classification accuracy without any pre-processing methods	[61]
VIS-NIR spectroscopy	Spectroradiometer	Healthy Mild Moderate Severe	LDA, QDA, kNN and NB	kNN has the highest classification performance: 97.3% accuracy Significant differences between each severity levels are observed in NIR region compared to VIS region	[46]
VIS-NIR spectroscopy	Spectroradiometer	Healthy Mild Moderate Severe	PLS-DA	Almost 94% classification accuracy	[65]

**Table 3:** Diagnostic functional group of NIR absorptions [70].

Functional Group	Found in
Hydroxyl (OH)	Water/Moisture, Carbohydrates, Sugars, Alcohols, Glycols
Amino (NH_2_)	Proteins, Polymers, Dyes, Pharmaceuticals
Alkyl/Aryl (C-H) Aliphatic and Aromatic Hydrocarbons	Fats/Lipids, Fuels, Plastics, Polymers

**Table 4:** Accuracy range for detection of plant disease in different IR spectroscopy regions.

IR Spectroscopy Region	Accuracy Range for Detection of Plant Disease
VIS-NIR	66–90%
NIR	90–96%
MIR	79–92%

**Table 5:** Previous studies on plant disease detection using NIRS.

Plant	Disease	Instrument	Wavelength (nm)	Models/Algorithms	Significant Result	Ref
Agaricus bisporus	Fungal contamination	FT-NIR spectrometer	833–2500	PLS-DA	Fungal species: 99% classification accuracy Storage period: 99.2% classification accuracy	[87]
Papaya	Begomovirus infection	NIR spectrophotometer	1000–2500	PLS-DA	Calibration: R^2^ = 0.964 Validation: R^2^ = 0.957	[79]
Potato	Zebra chip disease	NIR spectrophotometer	900–2600	Canonical DA	Raw spectra: 98.35% classification accuracy 2nd derivative spectra: 97.25% classification accuracy	[88]
Wheat	Stripe rust	FT-NIR spectrometer	833–2500	QPLS, SVR, QPLS+SVR	R^2^ > 0.5 for all models	[89]
Honeycrisp apple	Bitter pit	Spectroradiometer	800–2500	QDA, SVM	QDA: 73–96% classification accuracy SVM: 69–89% classification accuracy	[90]
Mango	Anthracnose disease	FT-NIR spectrometer	900–2500	PLS-DA	89% classification accuracy	[91]
Mango	Fruit fly eggs and larval infestation	NIRGun and the Bran + Luebbe InfraAlyzer 500	700–950 1100–2500	PLS-DA	700–950 Infested fruit: SD = 0.27 Control fruit: SD = 0.19 1100–2500 Infested fruit: SD = 0.26 Control fruit: SD = 0.28	[92]
Maize	Fungal infection	NIR spectrometer	500–1700	kNN	Healthy kernels: 98.1% classification accuracy Infected kernels: 96.6% classification accuracy	[93]
Maize	Fusarium infection	NIR spectrophotometer	400–2500	SIMCA, PNN, k-means	PNN has the best performance Healthy grain: 99.3% classification accuracy Infected grain: 98.7% classification accuracy	[94]
Maize	Fungal infection	NIR spectrometer	904–1685	LDA, MLP neural networks	Uninfected control kernels: 89% classification accuracy Infected kernels: 79% classification accuracy	[95]
Chestnut	Fungal infection	NIR analyser	1100–2300	LDA, QDA, kNN	The highest overall classification using QDA: 97% accuracy	[96]
Barley	Fusarium infection	NIR spectrometer	1175–2170	PLS-DA	Up to 100% classification accuracy	[80]
Almond	Fungal infection	VIS-NIR spectrophotometer	800–2500	Canonical DA	Cross-validation error rate = 0.26% False negative error = 0	[67]
Tomato	Leaf miner infestation	FT-NIR spectrometer	800–2500	Regression analysis	R^2^ = 0.982	[97]
Sugarcane	Fiji leaf gall	NIR spectrometer	909–2500	PLS	SEV = 0.98 (R^2^ = 0.97) SEP =1.20 (R^2^ = 0.88)	[98]
Rice	Aflatoxin B1 contamination	FT-NIR spectrometer	1000–2500	PLS	Correlation, R = 0.850, SEP = 3.211%	[99]
Red paprika	Aflatoxin B1 and ochratoxin A contamination	NIR spectrophotometer	1100–2000	MPLS	AFB_1_: R^2^ = 0.95 OTA: R^2^ = 0.85 Total aflatoxins: R^2^ = 0.93	[100]

**Table 6:** Advantages and disadvantages of classifiers.

Classifier	Advantages	Disadvantages
kNN	Simple implementation Classes do not have to be linearly separable	Sensitive to noisy or irrelevant data Testing procedure is time-consuming because of calculation of distance to all known instances
NB	Only a small amount of training data is required Has better speed	It cannot learn interactions between different features because dependency exists among variables
Decision tree	Easy to interpret for small trees Accuracy is comparable to other classification techniques for many simple datasets	Decision tree has been observed to overfit for some datasets with noisy classification tasks Restricted to one output attribute Complex decision tree for numeric datasets
ANN	Robust and user friendly and can handle noisy data Well suited to analysing complex problems	Scalability problem Requires large number of training samples Requires more processing time
SVM	Effective and robust to noise Highly accurate Can handle many features	Not suitable for large datasets Speed is slow and requires more time to process

## Data Availability

Not applicable.
